# Biologically integrated EGCG-modified palladium nanozyme for synergistic photothermal-catalytic therapy of esophageal cancer

**DOI:** 10.3389/fphar.2026.1862169

**Published:** 2026-07-24

**Authors:** Yuhang Shang, Yujie Zhao, Qi Li, Di Ai, Na An, Zhen Han, Sheng Zhang, Ziyi Li, Xinglan An

**Affiliations:** 1 Key Laboratory of Organ Regeneration and Transplantation of the Ministry of Education, The First Hospital of Jilin University, Jilin University, Changchun, China; 2 Department of Intensive Care Unit, The First Affiliated Hospital of Jiamusi University, Jiamusi, China

**Keywords:** biological integration, enzyme-mimicking activity, esophageal cancer, Pd-based nanozyme, photothermal therapy

## Abstract

**Introduction:**

Photothermal therapy (PTT) faces limitations due to tumor microenvironment (TME) heterogeneity and single-modality constraints, including hypoxia, redox imbalance, and uneven heat distribution, which compromise therapeutic durability. Integrating nanozyme catalysis with PTT presents a promising strategy to amplify oxidative stress, yet achieving a balance among catalytic efficiency, photothermal performance, biocompatibility, and stability remains challenging.

**Methods:**

Herein, we developed an epigallocatechin gallate (EGCG)-modified palladium-based nanozyme (EGCG-PdZyme) for the precision treatment of esophageal cancer. This multifunctional platform was engineered to integrate catalase-like oxygen generation, peroxidase-like reactive oxygen species (ROS) production, and near-infrared photothermal conversion capabilities.

**Results:**

While EGCG modification slightly attenuated the intrinsic catalytic activity and peak photothermal temperature, it established an optimized thermo-catalytic synergy. Sustained mild hyperthermia amplified oxidative stress, effectively offsetting the reduced catalytic output and minimizing thermal damage to peritumoral tissues. Mechanistically, persistent photothermal heating boosted enzymatic ROS generation within the TME, initiating a self-amplifying therapeutic cascade. Furthermore, EGCG functionalization significantly enhanced colloidal stability and biosafety, enabling effective tumor ablation with negligible systemic toxicity.

**Discussion:**

This study demonstrates a paradigm shift from maximizing isolated parameters toward achieving a dynamic equilibrium between catalytic functionality and biological compatibility. By integrating TME modulation with controlled photothermal amplification, the EGCG-PdZyme platform offers a viable strategy for clinically translatable precision oncotherapy.

## 1 Introduction

Esophageal cancer as a malignant tumor originates in the epithelial tissue of the esophagus. It is the eighth most common cancer globally as well as the sixth leading cause of cancer death (Chen et al., 2023). It is mainly divided into two histological subtypes: adenocarcinoma (AC) and squamous cell carcinoma (SCC) (Deboever et al., 2024; Yang et al., 2024). Due to the lack of obvious clinical symptoms in the early stages, most patients are diagnosed at an advanced stage with distant metastasis. The overall 5-year survival rate is low, ranging from 10% to 30%. The main treatment method in clinic for esophageal cancer include surgery, chemotherapy, immunotherapy, and targeted therapy (Waters and Reznik, 2022; Xin et al., 2023). However, since most esophageal cancer patients are diagnosed at an advanced stage with metastasis, the treatment outcome is not satisfied while side effects from radiotherapy and chemotherapy are obvious (Yang et al., 2023).

**SCHEME 1:**
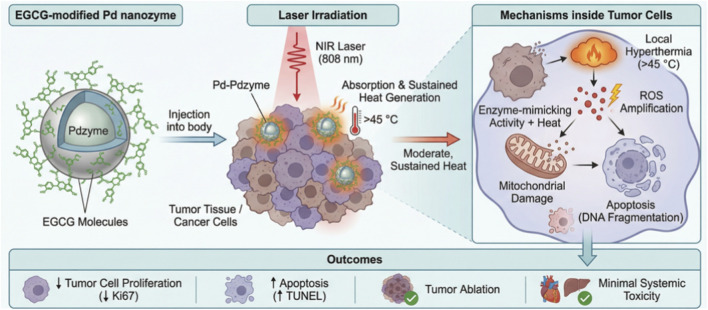
Graphical illustration of EGCG-Pdzyme mediated photothermal therapy integrating sustained hyperthermia and enzymatic effects for efficient tumor ablation.

As a non-invasive tumor treatment method, photothermal therapy (PTT) are showing potential in tumor ablation by converting light energy into heat with the assistance of photothermal transform agents (PTAs) (Cai et al., 2025). When local heat in tumor site exceeded 42 °C, severe damage was triggered by protein denaturation, cell membrane damage, mitochondrial damage and other methods to suppress tumor cell (Zhu et al., 2024; Gu et al., 2025). With specific tumor targeting and low side effects, PTT could be applied for local solid tumor which can not suffer surgery. Inorganic nanomaterials including gold, platinum, bismuth, ruthenium and other noble metals are developed for high-efficient photothermal conversion application of tumor suppression. Li’s team reported an iron-doped bismuth sulfide nanomaterials for triple-negative breast cancer treatment by mild PTT (Xie et al., 2025). An armored core-gold nanostar was synthesized by Vo-Dinh’s team to realize real-time imaging-guided PTT (Canning et al., 2026). Additionally, certain organic nanocomplexes exhibit immense potential as PTAs. Wang’s group developed a Si-cyclopentadithiophene-based organic molecule for tumor ablation under 808 nm laser irradiation (Fan et al., 2025). Notably, PTT often triggers a heat shock response (HSR) in tumor cells, which heightens their thermotolerance and ultimately impairs the therapeutic efficacy of PTT.

The HSR is a protective response initiated by cells under high-temperature stress, involving a series of molecular events primarily aimed at repairing protein misfolding, abnormal aggregation, and dysfunction caused by heat stress (Schroeder et al., 2024). A crucial part of this response is the activation of heat shock proteins (HSPs), which play a crucial role in assisting tumor cells adapt to high temperatures. This HSPs includes main families such as HSP70, HSP90, and HSP60, each of which performs specific functions. HSP70 assists in the folding, repair, and degradation of unstable proteins; HSP90 maintains normal cellular activity by stabilizing key signaling molecules; and HSP60 participates in mitochondrial protein folding to maintain the stability of the mitochondrial environment (Genest et al., 2019; Singh et al., 2024). Given the crucial support that HSP contributed to tumor cell survival under high-temperature conditions, inhibiting HSP expression has become one of the effective strategies for reducing tumor cell heat resistance and enhancing the efficacy of PTT (Sha et al., 2023). Recent research reported that oxidative stress can interfere with the activation of heat shock factor 1 or directly degrade HSPs proteins, becoming important pathways for inhibiting the expression of HSP70 and HSP90 (Zhang and Qi, 2025; Albakova et al., 2022). This provides key insights for optimizing photothermal therapy strategies.

Palladium-based nanozymes have attracted increasing attention in tumor therapy owing to their excellent catalytic activity, broad optical absorption, photothermal conversion capability, and physicochemical stability (Wang et al., 2025; Fang et al., 2026; Deshwal and Tripathi, 2025). Although Pd-based nanozymes possess intrinsic catalytic activity and near-infrared photothermal conversion capability, their therapeutic performance in biological systems is not solely determined by these physicochemical properties. The surface biointerface of nanomaterials plays a crucial role in colloidal stability, biocompatibility, tumor cell interaction, and therapeutic responsiveness. Epigallocatechin gallate (EGCG), a natural tea polyphenol, contains abundant phenolic hydroxyl groups and aromatic structures, which can interact with metal-based nanomaterials through coordination, hydrogen bonding, and π-related interactions (Marín et al., 2023). Moreover, EGCG has been reported to exhibit favorable biocompatibility and intrinsic anticancer activities, including regulation of oxidative stress, inhibition of tumor cell proliferation, and promotion of apoptosis (Li et al., 2024; Capasso et al., 2025). Therefore, EGCG modification is not intended simply to maximize the catalytic or photothermal performance of Pdzyme, but to introduce a bioactive polyphenolic interface that enables better biological integration.

In this study, we developed an EGCG-modified Pd-based nanozyme, termed EGCG-Pdzyme, for biologically integrated photothermal-catalytic therapy of esophageal cancer. Different from conventional nanozyme-polyphenol hybrids that mainly focus on material stabilization or catalytic regulation, this platform was designed to integrate Pdzyme-mediated catalytic activity, NIR-triggered photothermal ablation, and EGCG-enabled biological interface modulation within a single therapeutic system. EGCG modification endows the Pdzyme with a bioactive polyphenolic interface, which may improve compatibility with biological environments and contribute to tumor cell growth inhibition and apoptosis sensitization. As a result, EGCG-Pdzyme provides a coordinated therapeutic strategy in which photothermal heating and nanozyme-mediated catalytic regulation act together to enhance antitumor efficacy against esophageal cancer.

## 2 Result and discussion

### 2.1 Structural characteristics of Pdzyme and EGCG-Pdzyme

**FIGURE 1:**
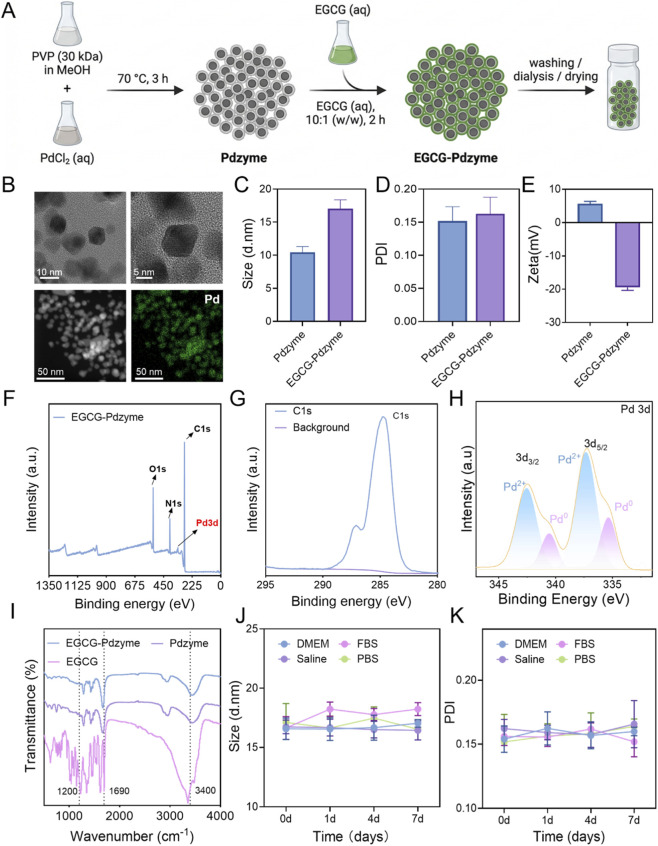
Fabrication and characterization Pdzyme and EGCG-Pdzyme. **(A)** Schematic illustration of the preparation of EGCG-Pdzyme. Pdzyme was first synthesized by reacting PdCl_2_ with polyvinylpyrrolidone (PVP) in methanol under heating, followed by the adsorption of epigallocatechin gallate (EGCG) to form EGCG-Pdzyme. The final product was purified by washing, dialysis, and drying. **(B)** TEM images and EDS mapping images of EGCG-Pdzyme. **(C–E)** Hydrodynamic diameter **(C)**, polydispersity index (PDI) **(D)**, and zeta potential **(E)** of Pdzyme and EGCG-Pdzyme. **(F)** Survey X-ray photoelectron spectroscopy (XPS) spectrum of EGCG-Pdzyme. **(G)** High-resolution XPS spectrum of the C 1s region. **(H)** High-resolution XPS spectrum of the Pd 3d region. **(I)** Fourier transform infrared (FTIR) spectra of EGCG, Pdzyme, and EGCG-Pdzyme. **(J,K)** Stability evaluation of EGCG-Pdzyme in different media (DMEM, FBS, saline, and PBS) over 7 days, showing changes in hydrodynamic diameter **(J)** and PDI **(K)**, demonstrating good colloidal stability.

EGCG-loaded Pdzyme nanozymes (EGCG-Pdzyme) were successfully constructed via a mild and controllable two-step synthesis strategy, as illustrated in Figure 1A. Initially, Pdzyme nanoparticles were prepared through a PVP-assisted reduction of PdCl_2_ in methanol at elevated temperature. PVP served not only as a stabilizing agent to prevent particle aggregation but also as a soft template to regulate nanoparticle growth. Subsequently, epigallocatechin gallate (EGCG) was introduced to the Pdzyme system, allowing surface functionalization through coordination interactions and hydrogen bonding between phenolic hydroxyl groups and Pd species. After purification by washing and dialysis, well-dispersed EGCG-Pdzyme nanoparticles were obtained.

Transmission electron microscopy (TEM) images revealed that pristine Pdzyme nanoparticles exhibited a uniform spherical morphology with good dispersion (Figure 1B). High-resolution TEM further confirmed the nanoscale dimensions and compact structure of Pdzyme. After EGCG modification, no obvious aggregation or morphological deformation was observed, indicating that surface functionalization did not compromise structural integrity. Dynamic light scattering (DLS) analysis showed that the hydrodynamic diameter of EGCG-Pdzyme increased compared with Pdzyme (Figure 1C), which can be attributed to the formation of an EGCG coating layer on the nanoparticle surface.

Both Pdzyme and EGCG-Pdzyme displayed low polydispersity index (PDI) values (<0.2), suggesting a narrow size distribution and good colloidal uniformity (Figure 1D). Such uniformity is particularly important for biomedical applications, as it contributes to predictable biological behavior and reproducible therapeutic performance. Notably, zeta potential measurements demonstrated a pronounced shift toward a more negative surface charge after EGCG modification (Figure 1E). This change originates from the abundant phenolic hydroxyl groups of EGCG and is expected to enhance colloidal stability via electrostatic repulsion, as well as influence biological interactions at the nano–bio interface.

X-ray photoelectron spectroscopy (XPS) was employed to further analyze the surface composition and chemical states of EGCG-Pdzyme. The survey spectrum confirmed the coexistence of C, O, N, and Pd elements (Figures 1F–H), consistent with the successful incorporation of organic EGCG molecules onto Pd-based nanozymes. As shown in Figure 1H, the Pd 3 days spectrum was fitted into two pairs of spin–orbit doublets. The peaks located at approximately 335.2 and 340.5 eV were assigned to Pd^0^ 3d_5_/_2_ and Pd^0^ 3d_3_/_2_, respectively, while the peaks at approximately 336.8 and 342.1 eV corresponded to Pd^2+^ 3d_5_/_2_ and Pd^2+^ 3d_3_/_2_, respectively. These results indicate the coexistence of metallic Pd and oxidized Pd species in EGCG-Pdzyme, which may contribute to its enzyme-like catalytic activity.

To further investigate the structural properties of Pdzyme and EGCG-Pdzyme, X-ray diffraction (XRD) analysis was performed (Supplementary Figure S1). Both samples exhibited broad diffraction patterns without sharp characteristic peaks, indicating a predominantly amorphous or poorly crystalline structure. Such structural features are commonly observed in ultrasmall metal-based nanozymes and are associated with high surface energy and abundant unsaturated active sites. As shown in Supplementary Figure S1B, Pdzyme and EGCG-Pdzyme displayed broad absorption from the UV to NIR region. Notably, EGCG-Pdzyme exhibited stronger absorbance than Pdzyme across 200–1,000 nm, including the NIR region, indicating its favorable photo-responsiveness.

Compared with pristine Pdzyme, EGCG-Pdzyme showed a slightly enhanced diffraction intensity at specific angles, suggesting that EGCG modification may subtly influence the local atomic arrangement of Pd species. However, no new crystalline phases or distinct diffraction peaks were detected, indicating that EGCG functionalization does not induce significant crystallization or phase transformation of Pdzyme.

The preservation of an amorphous Pd-based structure is advantageous for both catalytic and photothermal applications, as it facilitates efficient energy conversion and reactive site exposure. Therefore, the XRD results, in combination with microscopy and spectroscopic analyses, confirm that EGCG-Pdzyme maintains the intrinsic structural characteristics of Pdzyme while achieving improved surface properties and stability. In addition, The FTIR spectra were used to characterize the functional groups of EGCG, Pdzyme, and EGCG-Pdzyme. As shown in Figure 1I, EGCG exhibited characteristic absorption bands at approximately 3,400 cm^−1^, 1,690 cm^−1^, and 1,200 cm^−1^, which were attributed to O–H stretching, C=O stretching, and C–O/C–O–C stretching vibrations, respectively. Compared with free EGCG, the EGCG-Pdzyme spectrum retained these characteristic absorption features, although the peak intensity and/or position showed slight changes. These results suggest the successful incorporation of EGCG into Pdzyme and the possible interaction between EGCG and Pdzyme.

The colloidal stability of EGCG-Pdzyme was systematically evaluated in different physiological media, including PBS, saline, DMEM, and FBS. As shown in Figures 1J,K, both hydrodynamic size and PDI of EGCG-Pdzyme remained nearly unchanged over a 7-day period, indicating excellent stability under biologically relevant conditions. This stability is essential for ensuring reliable circulation behavior and therapeutic efficacy in subsequent *in vitro* and *in vivo* applications.

### 2.2 CAT- and POD-like enzymatic activities of EGCG-Pdzyme

**FIGURE 2:**
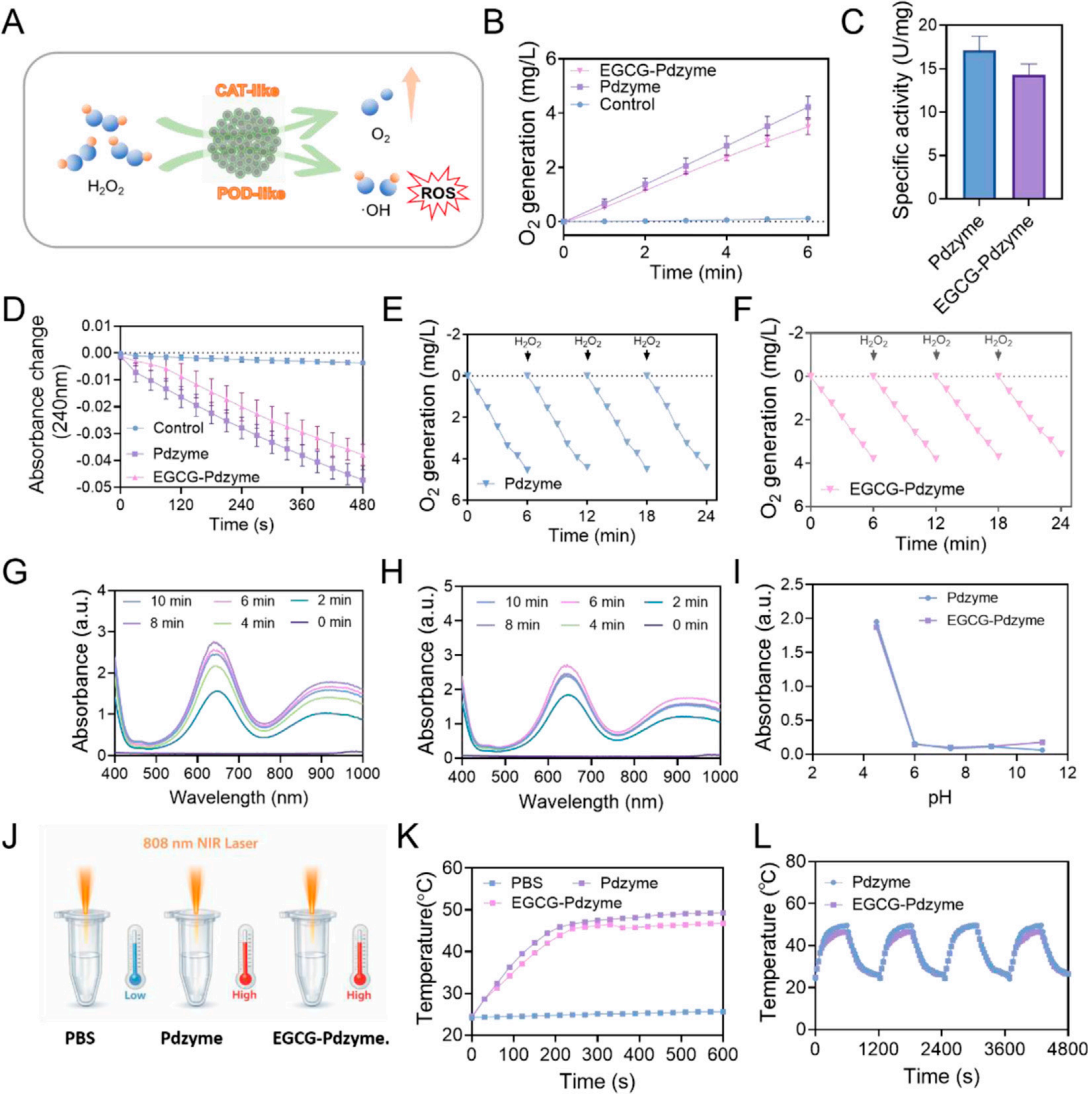
Catalytic activities and enzyme-mimicking properties of Pdzyme and EGCG-Pdzyme. **(A)** Schematic illustration of the catalytic activity conversion between catalase (CAT)-like and peroxidase (POD)-like activities. **(B)** CAT-like activity of Pdzyme and EGCG-Pdzyme by O_2_ production over time **(C)** Specific catalytic activities of Pdzyme and EGCG-Pdzyme, expressed as units per milligram. **(D)** H_2_O_2_ degradation after treatment with PBS, Pdzyme and EGCG-Pdzyme at different time points. **(E,F)** Continuous H_2_O_2_ consumption after treatment with Pdzyme and EGCG-Pdzyme by repetitive addition of H_2_O_2_. **(G)** POD-like catalytic activity of Pdzyme was evaluated by full-wavelength scanning spectroscopy, and the time-dependent absorbance changes were monitored to assess its peroxidase-mimicking performance. **(H)** POD-like catalytic activity of EGCG-Pdzyme was evaluated by full-wavelength scanning spectroscopy, and the time-dependent absorbance changes were monitored to assess its peroxidase-mimicking performance. **(I)** Under different pH conditions, using TMB as the detection substrate, the absorbance changes of the Pdzyme and EGCG-Pdzyme reaction system at 652 nm. **(J)** Schematic illustration of temperature changes of different samples upon 808 nm NIR laser irradiation. **(K)** Temperature elevation curves of PBS, Pdzyme, and EGCG-Pdzyme under continuous 808 nm NIR laser irradiation (1.2 W/cm^2^). **(L)** Temperature variation profiles of Pdzyme and EGCG-Pdzyme during repeated laser on/off cycles, demonstrating photothermal stability.

The enzyme-mimicking catalytic activities of Pdzyme and EGCG-Pdzyme were systematically evaluated, focusing on their catalase-like (CAT-like) and peroxidase-like (POD-like) behaviors. As illustrated schematically in Figure 2A, Pd-based nanozymes are capable of catalyzing hydrogen peroxide–related reactions, enabling oxygen generation and substrate oxidation.

The CAT-like activity of Pdzyme and EGCG-Pdzyme was first assessed by monitoring oxygen (O_2_) generation from hydrogen peroxide (H_2_O_2_). As shown in Figure 2B, both Pdzyme and EGCG-Pdzyme induced continuous oxygen evolution over time, while negligible O_2_ generation was observed in the control group. Notably, Pdzyme exhibited a slightly higher oxygen generation rate than EGCG-Pdzyme, indicating that EGCG modification partially attenuates the intrinsic CAT-like activity of Pdzyme. The specific CAT-like activity was further quantified and normalized by catalyst mass (Figure 2C). Consistent with the oxygen generation results, EGCG-Pdzyme displayed a modestly lower specific activity compared with pristine Pdzyme. This slight decrease can be attributed to partial surface coverage of Pd active sites by EGCG molecules, which may limit direct access of H_2_O_2_ to catalytic Pd centers. The decomposition of H_2_O_2_ was further confirmed by monitoring the decrease in absorbance at 240 nm (Figure 2D). Both Pdzyme and EGCG-Pdzyme caused a time-dependent decline in absorbance, with Pdzyme showing a faster reaction rate, further validating the reduced but preserved CAT-like activity after EGCG modification. The recyclability of CAT-like activity was evaluated through repeated H_2_O_2_ addition experiments (Figures 2E,F). Both Pdzyme and EGCG-Pdzyme maintained stable oxygen generation behavior over multiple cycles, demonstrating good catalytic durability. Although EGCG-Pdzyme showed slightly lower oxygen output per cycle, its sustained performance indicates that EGCG functionalization does not compromise catalytic stability.

Time-dependent visual observation of oxygen bubble formation further supported these results (Supplementary Figure S2). Compared with Pdzyme, EGCG-Pdzyme exhibited slightly reduced but clearly observable bubble generation, confirming retained CAT-like activity at the macroscopic level.

The influence of pH and temperature on CAT-like activity was further examined (Supplementary Figure S3). Both Pdzyme and EGCG-Pdzyme exhibited maximal activity at physiological pH (∼7.4) and body temperature (37 °C). Although EGCG-Pdzyme showed consistently lower activity values across different conditions, the overall trends were similar, indicating that EGCG modification does not alter the fundamental catalytic behavior but slightly modulates reaction efficiency.

The peroxidase-like (POD-like) activity of Pdzyme and EGCG-Pdzyme was systematically investigated using both spectroscopic and chromogenic assays. Time-dependent UV–vis absorption spectra shown in Figures 2G,H reveal the gradual formation of oxidized products in the presence of Pdzyme and EGCG-Pdzyme, confirming their ability to catalyze POD-like reactions. Quantitative analysis of absorbance changes (Figure 2I) further demonstrated that EGCG-Pdzyme exhibited slightly lower POD-like activity compared with pristine Pdzyme. To further verify POD-like catalytic behavior, classical chromogenic substrates, including o-phenylenediamine (OPD) and 3,3′,5,5′-tetramethylbenzidine (TMB), were employed (Supplementary Figure S4). Both Pdzyme and EGCG-Pdzyme efficiently catalyzed substrate oxidation in the presence of H_2_O_2_, generating characteristic absorption peaks, whereas negligible signals were observed in the H_2_O control group. Consistent with previous results, EGCG-Pdzyme showed reduced absorbance intensity relative to Pdzyme, indicating a moderate decrease in POD-like activity after EGCG modification. The slight reduction in POD-like activity can be reasonably explained by the surface shielding effect of EGCG, which partially blocks Pd catalytic sites while simultaneously introducing biocompatible and stabilizing functionalities. Importantly, despite this decrease, EGCG-Pdzyme retains sufficient POD-like activity for effective ROS-related catalytic processes.

Collectively, these results demonstrate that EGCG modification leads to a mild reduction in both CAT- and POD-like activities of Pdzyme, primarily due to partial surface coverage of active Pd sites. However, EGCG-Pdzyme maintains stable, reproducible, and environmentally adaptable catalytic performance. Importantly, the trade-off between catalytic activity and improved surface properties, stability, and biocompatibility provides a rational basis for its application in photothermal-assisted cancer therapy.

In addition to enzyme-mimicking catalytic activity, the photothermal performance of Pdzyme and EGCG-Pdzyme was systematically investigated under near-infrared (NIR) laser irradiation. As shown in Figures 2J,K, both Pdzyme and EGCG-Pdzyme exhibited rapid temperature elevation upon NIR exposure, whereas negligible temperature change was observed in the PBS control group, confirming their effective photothermal conversion capability. Notably, EGCG-Pdzyme displayed a comparable, and in some cases slightly more stable, photothermal heating profile compared with pristine Pdzyme. Upon continuous laser irradiation, the temperature of EGCG-Pdzyme solutions increased sharply and reached therapeutically relevant levels within a short time period. To quantitatively evaluate the photothermal performance of EGCG-Pdzyme, its photothermal conversion efficiency was further calculated. As shown in Supplementary Figure S5, based on the linear fitting of the cooling period, the time constant (τs) was determined to be 194.91 s, and the photothermal conversion efficiency (η) was calculated to be 22.1%, indicating the good photothermal conversion capability of EGCG-Pdzyme. Moreover, repeated laser on/off cycles resulted in nearly identical temperature elevation curves, indicating excellent photothermal stability and resistance to photobleaching (Figure 2L). These results demonstrate that surface modification with EGCG does not compromise the intrinsic photothermal properties of Pd-based nanozymes. The strong photothermal performance of EGCG-Pdzyme can be attributed to the broad NIR absorption of Pd nanostructures, while the organic EGCG layer provides additional structural stability and dispersibility, facilitating efficient light-to-heat conversion in aqueous and biological environments.

It should be noted that EGCG modification slightly attenuated the intrinsic catalytic activity and photothermal heating performance of Pdzyme, which may be attributed to partial surface coverage of catalytically active Pd sites by the polyphenolic layer. However, the therapeutic efficacy of EGCG-Pdzyme cannot be simply evaluated by catalytic or photothermal parameters alone. EGCG provides a bioactive interface that improves the biological integration of Pdzyme and may contribute to enhanced tumor cell interaction, apoptosis sensitization, and favorable biosafety. Therefore, EGCG modification represents a trade-off between preserving sufficient catalytic/photothermal activity and introducing a biologically functional surface. The superior antitumor outcome of EGCG-Pdzyme under NIR irradiation suggests that the EGCG-enabled biological contribution synergizes with Pdzyme-mediated photothermal-catalytic effects.

Furthermore, EGCG itself possesses biological activity, including redox modulation and metal-chelating properties, which may further influence intracellular oxidative balance and sensitize tumor cells to photothermal stress. Therefore, EGCG-Pdzyme integrates moderate enzyme-mimicking activity, efficient photothermal conversion, and bioactive surface modulation into a single nanosystem.

Rather than relying solely on maximal catalytic activity, EGCG-Pdzyme achieves enhanced therapeutic efficacy through the synergistic integration of stable photothermal performance and retained enzyme-mimicking functions, making it particularly suitable for photothermal therapy of esophageal cancer.

### 2.3 *In vitro* and in vivo biosafety evaluation of EGCG-Pdzyme

The biosafety of EGCG-Pdzyme was systematically evaluated through a combination of *in vitro* and *in vivo* experiments, including hemolysis assays, cytocompatibility assessment, body weight monitoring, blood biochemical analysis, and histological examination of major organs.

**FIGURE 3:**
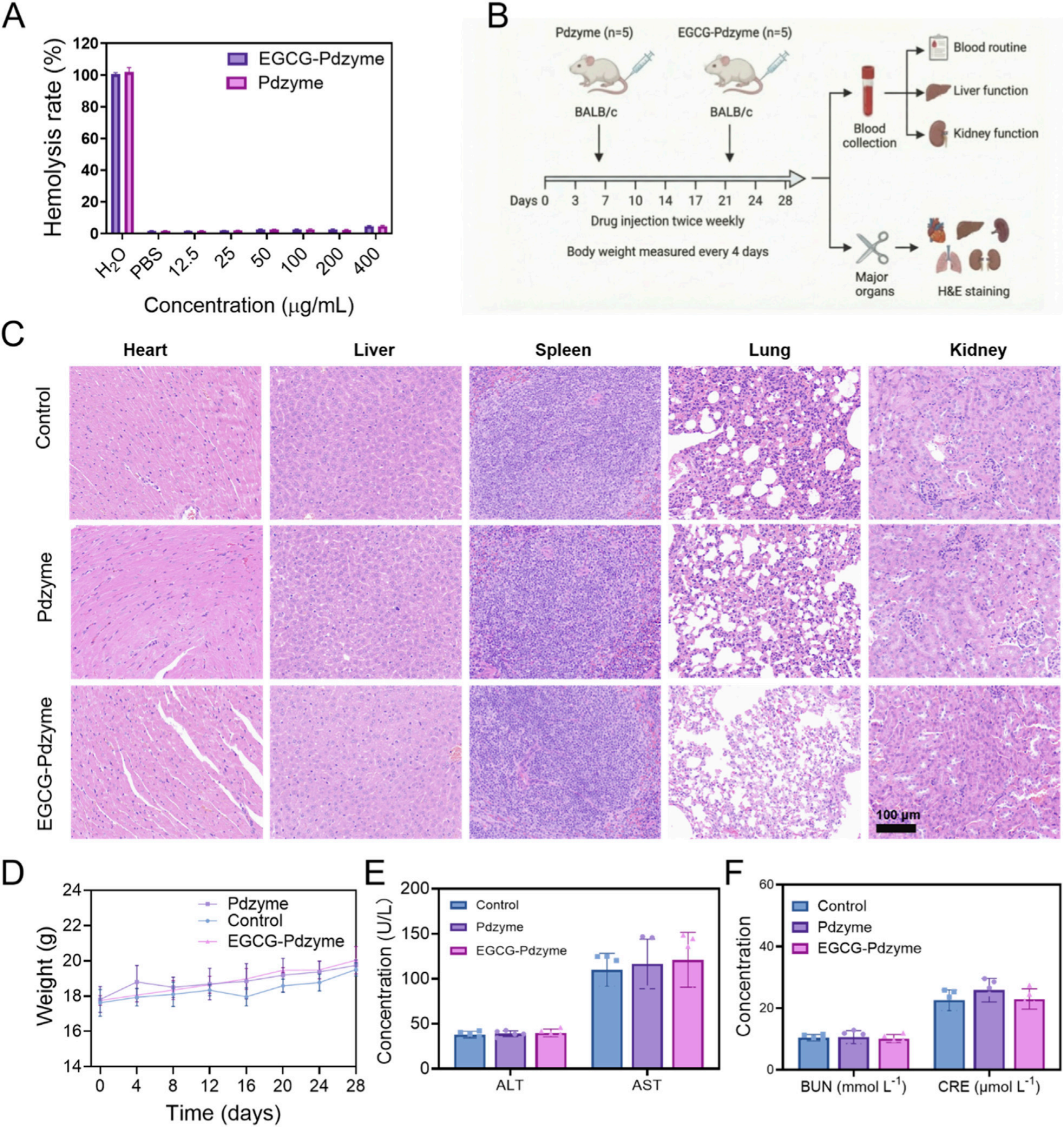
*In vitro* hemocompatibility and *in vivo* biosafety evaluation of Pdzyme and EGCG-Pdzyme. **(A)** Hemolysis assay of Pdzyme and EGCG-Pdzyme at different concentrations, with PBS and deionized water (H_2_O) used as negative and positive controls, respectively. **(B)** Schematic illustration of the *in vivo* biosafety study design in BALB/c mice (n = 5 per group). **(C)** Representative H&E-stained sections of major organs (heart, liver, spleen, lung, and kidney) from control, Pdzyme-treated, and EGCG-Pdzyme-treated mice. Scale bar: 100 μm. **(D)** Body weight changes of mice during the 28-day treatment period. **(E)** Serum biochemical parameters for liver function evaluation, including alanine aminotransferase (ALT) and aspartate aminotransferase (AST). **(F)** Serum biochemical parameters for kidney function evaluation, including blood urea nitrogen (BUN) and creatinine (CRE).

Hemocompatibility is a critical prerequisite for nanomaterials intended for biomedical applications. As shown in Figure 3A, both Pdzyme and EGCG-Pdzyme induced negligible hemolysis over a wide concentration range, even at high concentrations up to 400 μg/mL. The hemolysis rates were comparable to that of the PBS control and were significantly lower than the positive control (H_2_O), indicating excellent blood compatibility.

The cytocompatibility of EGCG-Pdzyme was evaluated using three representative normal cell lines, including LX2, AC16, and HEK293 cells. As shown in Supplementary Figure S6, cell viability remained above 80% after incubation with EGCG-Pdzyme over a wide concentration range (12.5–200 μg/mL) for both 24 and 48 h. Although a slight decrease in cell viability was observed at higher concentrations and longer incubation times, no significant cytotoxicity was detected. These results demonstrate that EGCG-Pdzyme exhibits good cytocompatibility toward normal cells, supporting its suitability for further biomedical applications.

To further evaluate *in vivo* biosafety, BALB/c mice were intravenously injected with Pdzyme or EGCG-Pdzyme according to the experimental design illustrated in Figure 3B. During the 28-day observation period, body weight was recorded every 4 days. As shown in Figure 3D, no significant differences in body weight were observed among the control, Pdzyme, and EGCG-Pdzyme groups, suggesting that repeated administration did not induce systemic toxicity or impair normal growth.

At the end of the treatment period, blood samples were collected for liver and kidney function analysis. As shown in Figures 3E,F, serum levels of alanine aminotransferase (ALT), aspartate aminotransferase (AST), blood urea nitrogen (BUN), and creatinine (CRE) in the Pdzyme- and EGCG-Pdzyme-treated groups were comparable to those in the control group. These results indicate that neither Pdzyme nor EGCG-Pdzyme caused obvious hepatic or renal dysfunction.

Histological analysis of major organs, including the heart, liver, spleen, lung, and kidney, was further performed by hematoxylin and eosin (H&E) staining (Figure 3C). No noticeable pathological abnormalities, inflammatory infiltration, or tissue damage were observed in the treated groups compared with the control group, confirming good *in vivo* biocompatibility.

To further assess systemic biosafety, routine hematological parameters were analyzed after treatment. As shown in Supplementary Figure S7, key blood indicators related to immune function (WBC, Lym, Mon), erythrocytes (RBC, HGB, HCT, MCV, MCH, MCHC), and platelets (PLT, MPV, PDW, PCT) in the Pdzyme and EGCG-Pdzyme groups were comparable to those of the control group. Although minor fluctuations were observed among different parameters, all values remained within normal physiological ranges, indicating that neither Pdzyme nor EGCG-Pdzyme induced significant hematological toxicity.

Collectively, the results from hemolysis assays, cytocompatibility tests, body weight monitoring, blood biochemical analysis, hematological evaluation, and histological examination demonstrate that EGCG-Pdzyme exhibits favorable biocompatibility both *in vitro* and *in vivo*.

### 2.4 *In vitro* antitumor effect of EGCG-Pdzyme

The *in vitro* antitumor efficacy of EGCG-Pdzyme was evaluated using esophageal cancer cells under different treatment conditions, including Pdzyme, EGCG-Pdzyme, laser irradiation, and their combinations. Cell viability, intracellular ROS generation, and apoptosis were systematically analyzed to elucidate the therapeutic mechanism.

**FIGURE 4:**
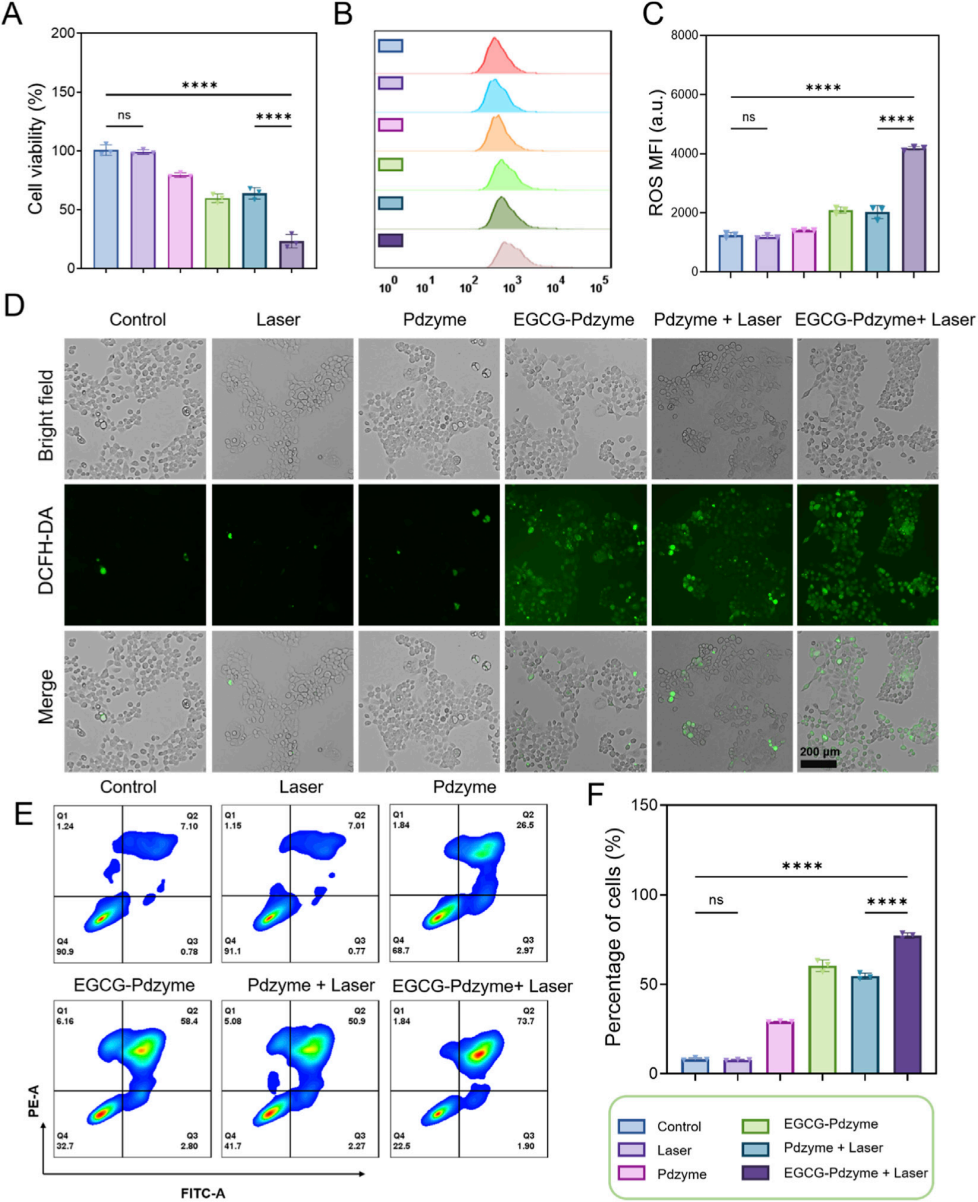
Photodynamic therapeutic effects of Pdzyme and EGCG-Pdzyme *in vitro*. **(A)** Cell viability of cells after different treatments. **(B)** Flow cytometry histograms showing intracellular ROSlevels under various treatment conditions. **(C)** Quantification of ROS generation expressed as mean fluorescence intensity (MFI), indicating enhanced ROS production in the Pdzyme + Laser and EGCG-Pdzyme + Laser groups. **(D)** Representative fluorescence microscopy images of intracellular ROS stained with DCFH-DA. Green fluorescence intensity increased markedly after laser irradiation in the nanoparticle-treated groups. Scale bar: 200 μm. **(E)** Flow cytometry analysis of apoptosis using Annexin V-FITC/PI staining after different treatments. **(F)** Percentage of early apoptotic cells (Q2 quadrant) quantified from Annexin V/PI analysis, demonstrating that EGCG-Pdzyme combined with laser irradiation induces the highest apoptotic response.

As shown in Figure 4A, treatment with laser irradiation alone or Pdzyme/EGCG-Pdzyme alone did not induce significant cytotoxicity, indicating minimal intrinsic toxicity of the nanozymes and laser at the applied conditions. In contrast, both Pdzyme + Laser and EGCG-Pdzyme + Laser groups exhibited a pronounced reduction in cell viability, demonstrating effective photothermal-triggered cytotoxicity. Notably, EGCG-Pdzyme combined with laser irradiation resulted in the lowest cell viability among all groups, suggesting an enhanced antitumor effect.

To further investigate the underlying mechanism, intracellular ROS levels were analyzed by flow cytometry using a DCFH-DA probe. As shown in Figure 4B, cells treated with EGCG-Pdzyme + Laser displayed a significant rightward shift in fluorescence intensity compared with other groups, indicating markedly increased ROS production. Quantitative analysis of mean fluorescence intensity (MFI) further confirmed that ROS levels in the EGCG-Pdzyme + Laser group were significantly higher than those in the Pdzyme + Laser group (Figure 4C). Fluorescence microscopy observations further supported these findings. As shown in Figure 4D, strong green fluorescence signals corresponding to ROS generation were observed in cells treated with EGCG-Pdzyme + Laser, whereas weak or negligible fluorescence was detected in control, laser-only, or nanozyme-only groups. These results demonstrate that laser irradiation effectively activates EGCG-Pdzyme to induce intracellular oxidative stress.

Apoptosis and cell death were further analyzed by Annexin V/PI staining followed by flow cytometry. Representative dot plots (Figure 4E) revealed a substantial increase in both early and late apoptotic cell populations in the EGCG-Pdzyme + Laser group. Quantitative analysis (Figure 4F) showed that the total apoptotic rate in the EGCG-Pdzyme + Laser group was significantly higher than that in the Pdzyme + Laser group, confirming enhanced tumor cell killing efficiency.

Collectively, these results demonstrate that EGCG-Pdzyme exhibits potent *in vitro* antitumor activity upon laser irradiation. The enhanced therapeutic effect can be attributed to the synergistic combination of photothermal-induced cellular damage and ROS-mediated oxidative stress. Although EGCG-Pdzyme shows slightly reduced intrinsic enzyme-like activity compared with Pdzyme, its superior photothermal performance and effective ROS amplification under laser stimulation result in improved overall antitumor efficacy.

### 2.5 *In vivo* antitumor efficacy of EGCG-Pdzyme–mediated photothermal therapy

The *in vivo* antitumor efficacy of EGCG-Pdzyme–mediated photothermal therapy was further evaluated using an esophageal cancer xenograft mouse model. Tumor-bearing mice were randomly divided into six groups, including Control, Laser, Pdzyme, EGCG-Pdzyme, Pdzyme + Laser, and EGCG-Pdzyme + Laser, and the therapeutic outcomes were systematically assessed in terms of survival rate, tumor growth, and final tumor burden.

**FIGURE 5:**
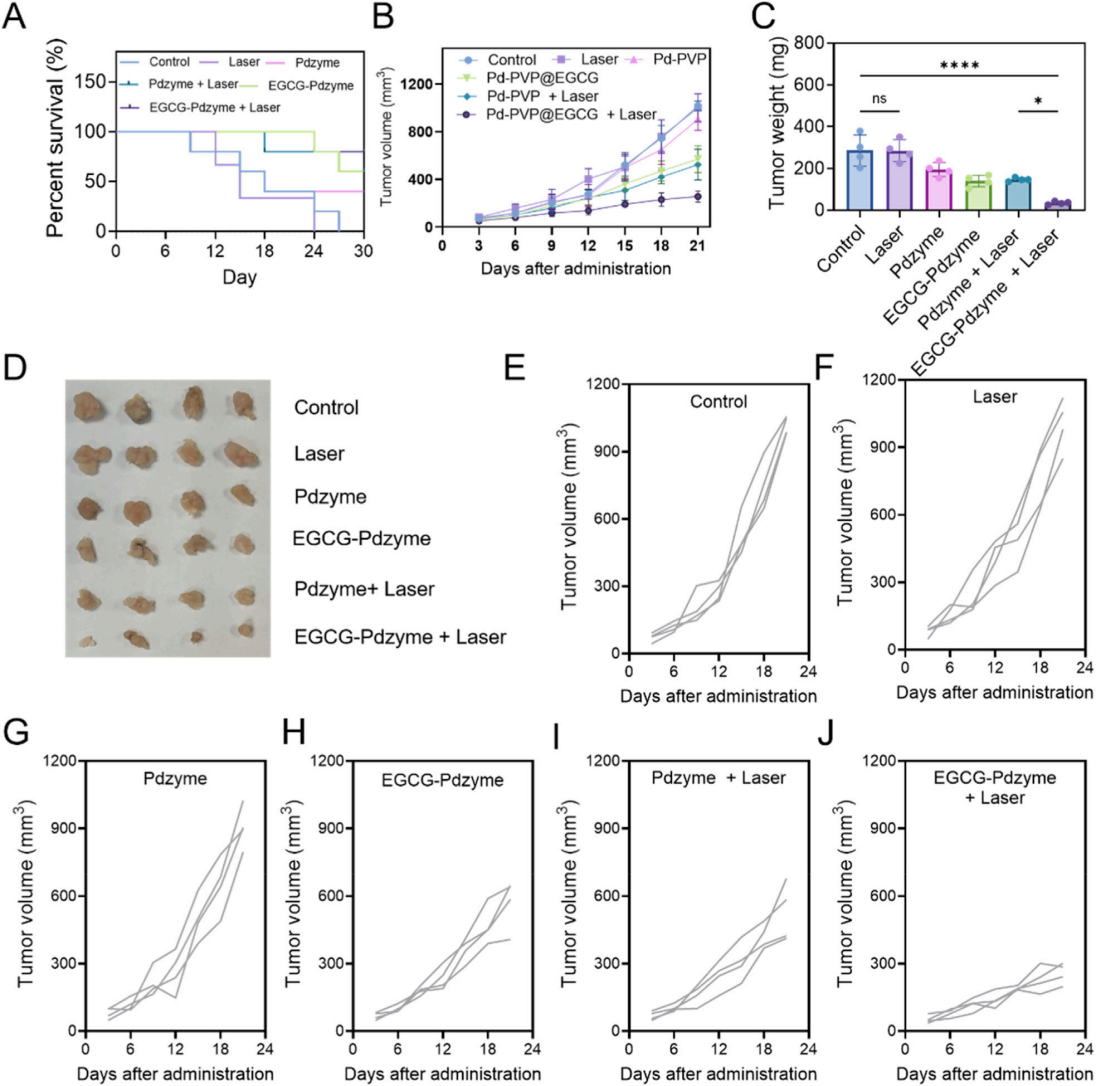
*In vivo* antitumor efficacy of Pdzyme- and EGCG-Pdzyme–mediated phototherapy. **(A)** Kaplan–Meier survival curves of tumor-bearing mice after different treatments. **(B)** Tumor growth curves showing the change in tumor volume over time after various treatments. **(C)** Tumor weights collected at the end of the treatment period. Data are presented as mean ± SD, with statistical significance indicated (*p < 0.05, ****p < 0.0001; ns, not significant). **(D)** Representative photographs of excised tumors from different treatment groups at the experimental endpoint. **(E–J)** Individual tumor volume growth curves for mice in each treatment group: Control **(E)**, Laser **(F)**, Pdzyme **(G)**, EGCG-Pdzyme **(H)**, Pdzyme + Laser **(I)**, and EGCG-Pdzyme + Laser **(J)**, demonstrating inter-animal variability and treatment efficacy.

As shown in Figure 5A, Kaplan–Meier survival analysis revealed that mice treated with EGCG-Pdzyme combined with laser irradiation exhibited the highest survival rate over the 30-day observation period. In contrast, mice in the control, laser-only, Pdzyme-only, and EGCG-Pdzyme-only groups showed limited survival benefits, indicating that neither laser irradiation nor nanozyme treatment alone was sufficient to achieve effective tumor control. Although the Pdzyme + Laser group showed improved survival compared with monotherapy groups, the survival advantage was markedly inferior to that of the EGCG-Pdzyme + Laser group, highlighting the superior therapeutic efficacy of EGCG-Pdzyme–mediated photothermal therapy.

Tumor growth curves further demonstrated the antitumor effects of different treatments (Figure 5B). Tumors in the control and monotherapy groups exhibited rapid and continuous growth throughout the treatment period. In contrast, tumor growth was significantly suppressed in the Pdzyme + Laser group, while the EGCG-Pdzyme + Laser group showed the most pronounced tumor inhibition, with minimal tumor volume increase over time. These results indicate that EGCG-Pdzyme, when activated by laser irradiation, effectively inhibits tumor progression *in vivo*.

At the end of the treatment period, tumors were excised and weighed to further quantify therapeutic outcomes. As shown in Figure 5C, the average tumor weight in the EGCG-Pdzyme + Laser group was significantly lower than that in all other groups, consistent with the tumor volume measurements. Representative photographs of excised tumors (Figure 5D) visually confirmed that tumors from the EGCG-Pdzyme + Laser group were substantially smaller than those from the other treatment groups.

To further evaluate the consistency and reliability of treatment efficacy, individual tumor growth curves were analyzed for each mouse (Figures 5E–J). Mice treated with EGCG-Pdzyme + Laser exhibited uniformly suppressed tumor growth with minimal interindividual variability, whereas tumors in the control and monotherapy groups displayed heterogeneous and rapid growth patterns. This consistency highlights the robustness and reproducibility of EGCG-Pdzyme–mediated photothermal therapy *in vivo*.

Taken together, these *in vivo* results demonstrate that EGCG-Pdzyme combined with laser irradiation achieves superior antitumor efficacy compared with Pdzyme-based photothermal therapy alone. The enhanced therapeutic outcome can be attributed to the synergistic effects of efficient photothermal ablation and retained enzyme-mimicking activity, which together induce irreversible tumor damage. Importantly, despite the slightly reduced intrinsic catalytic activity of EGCG-Pdzyme, its improved stability, biocompatibility, and photothermal performance result in a more effective and durable antitumor response *in vivo*.

### 2.6 *In vivo* photothermal performance and histological mechanism validation

To further validate the *in vivo* photothermal performance and therapeutic mechanism of EGCG-Pdzyme–mediated photothermal therapy, infrared thermal imaging and comprehensive histological analyses were conducted in tumor-bearing mice after different treatments.

**FIGURE 6:**
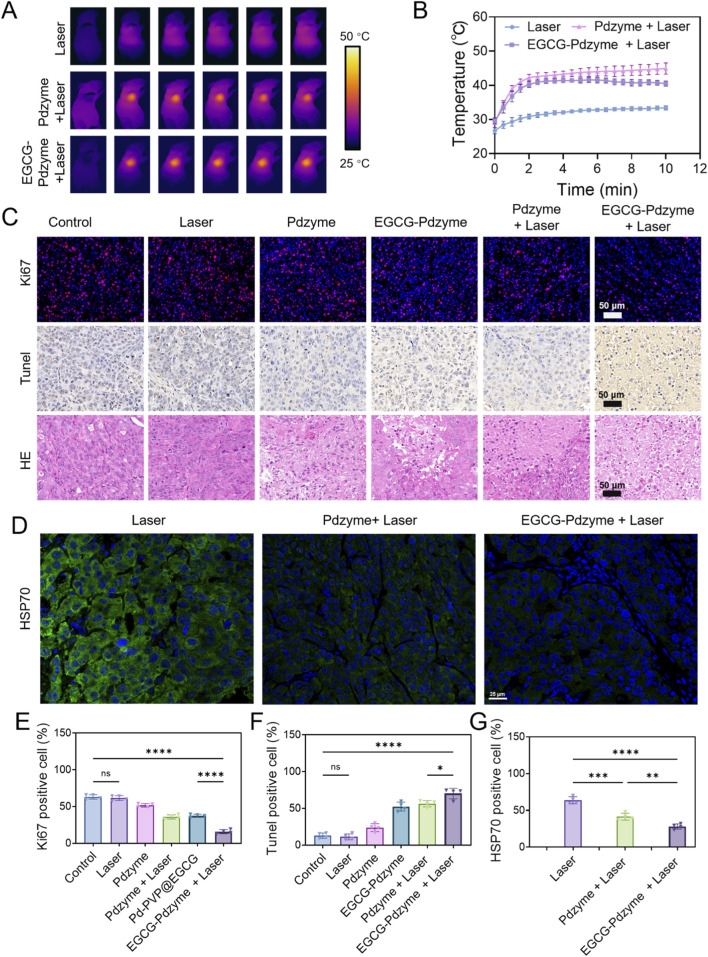
*In vivo* photothermal performance and histological evaluation after different treatments. **(A)** Representative infrared thermal images of tumor-bearing mice during laser irradiation in the Laser, Pdzyme + Laser, and EGCG-Pdzyme + Laser groups. **(B)** Temperature elevation curves of the tumor region during laser exposure. **(C)** Representative tumor tissue staining after various treatments. Ki67 staining was used to evaluate tumor cell proliferation, TUNEL staining reflected apoptosis, and H&E staining showed tissue morphology and damage. Scale bar: 50 μm. **(D)** Representative tumor tissue staining of HSP70 after various treatments. Scale bar: 25 μm. **(E–G)** Quantitative analysis of Ki67-positive cells **(E)**, TUNEL-positive cells **(F)**, and HSP70 expression **(G)** in tumor sections.

The photothermal heating behavior of tumors was first monitored by infrared thermal imaging during laser irradiation. As shown in Figure 6A, both Pdzyme + Laser and EGCG-Pdzyme + Laser groups exhibited rapid temperature elevation at the tumor site, whereas laser irradiation alone induced only a modest temperature increase. Quantitative temperature–time profiles further revealed that the Pdzyme + Laser group reached a slightly higher peak temperature compared with the EGCG-Pdzyme + Laser group (Figure 6B). Nevertheless, tumors treated with EGCG-Pdzyme + Laser were still efficiently heated into the therapeutic hyperthermia range (>45 °C), which is sufficient to induce irreversible tumor damage. These results indicate that although EGCG modification slightly attenuates peak photothermal heating, EGCG-Pdzyme retains effective *in vivo* photothermal conversion capability.

To assess therapeutic efficacy at the tissue level, tumor sections were subjected to hematoxylin and eosin (H&E) staining as well as immunohistochemical staining for Ki67, TUNEL, and HSP70 (Figure 6C). H&E staining revealed that tumors in the control, laser-only, Pdzyme, and EGCG-Pdzyme groups largely maintained intact tissue architecture. In contrast, tumors treated with Pdzyme + Laser showed evident tissue damage, while the EGCG-Pdzyme + Laser group exhibited more extensive tumor destruction, including pronounced cellular disorganization, nuclear fragmentation, and large necrotic regions, indicating more effective tumor ablation.

Ki67 immunostaining was used to evaluate tumor cell proliferation. As shown in Figures 6C,E, high levels of Ki67-positive cells were observed in the control and monotherapy groups. Both Pdzyme + Laser and EGCG-Pdzyme + Laser treatments significantly reduced Ki67 expression. Notably, the EGCG-Pdzyme + Laser group exhibited the lowest proportion of Ki67-positive cells, suggesting more effective suppression of tumor proliferation despite its slightly lower peak photothermal temperature.

Apoptosis within tumor tissues was assessed by TUNEL staining. As shown in Figures 6C,F, minimal apoptotic signals were detected in the control, laser-only, Pdzyme, and EGCG-Pdzyme groups. In contrast, both Pdzyme + Laser and EGCG-Pdzyme + Laser treatments markedly increased the number of TUNEL-positive cells, with the EGCG-Pdzyme + Laser group showing the highest apoptotic index. These results indicate that EGCG-Pdzyme–mediated photothermal therapy induces more extensive tumor cell apoptosis *in vivo*. The expression of HSP70, a marker of cellular stress response, was further analyzed. As shown in Figures 6D,G, laser irradiation alone induced elevated HSP70 expression, reflecting an adaptive stress response. In contrast, tumors treated with Pdzyme + Laser and EGCG-Pdzyme + Laser exhibited altered HSP70 expression patterns, suggesting that intense photothermal stress exceeded the protective capacity of tumor cells.

Collectively, these results demonstrate that EGCG-Pdzyme–mediated photothermal therapy achieves superior antitumor efficacy not by generating the highest peak temperature, but by inducing effective and sustained thermal stress that suppresses proliferation and promotes apoptosis. The slightly reduced peak temperature caused by EGCG modification represents a favorable trade-off for improved therapeutic performance and biological response *in vivo*.

## 3 Conclusion

In summary, we have developed an EGCG-Pdzyme as a multifunctional platform for photothermal therapy of esophageal cancer. EGCG-Pdzyme integrates dual enzyme-mimicking activities (CAT- and POD-like) with efficient near-infrared photothermal conversion, enabling a biologically coordinated therapeutic strategy.

This work underscores that therapeutic efficacy is governed by the rational integration of physicochemical functionality and biological responsiveness, rather than by maximizing catalytic activity or peak temperature alone. EGCG-Pdzyme exemplifies a balanced nanozyme design that harmonizes catalytic performance, photothermal efficiency, and biosafety, offering a promising strategy for precision photothermal oncology and providing conceptual guidance for the future development of multifunctional nanotherapeutics.

## Data Availability

The original contributions presented in the study are included in the article/Supplementary Material, further inquiries can be directed to the corresponding author.
