# Supplementary material for: Biologically integrated EGCG-modified palladium nanozyme for synergistic photothermal-catalytic therapy of esophageal cancer
Source: Front Pharmacol. 2026 Jul 24;17:1862169. doi: 10.3389/fphar.2026.1862169 (PMC13447497; doi:10.3389/fphar.2026.1862169)
Supplement: Supplementary file 1 [file Supplementaryfile1.docx]

**Experimental Section**

1. **Chemicals and Materials**

Epigallocatechin gallate (EGCG) was obtained from Aladdin (China). Polyvinylpyrrolidone (PVP, Mw = 30 kDa) was purchased from Sigma-Aldrich (USA). Palladium precursor (e.g., PdCl₂ or Na₂PdCl₄) was purchased from Sigma-Aldrich (USA). RPMI 1640 culture medium and fetal bovine serum (FBS) were purchased from Gibco (USA). Cell Counting Kit-8 (CCK-8) was obtained from APExBIO (USA). 2’,7’-Dichlorodihydrofluorescein diacetate (DCFH-DA) was purchased from Beyotime (China). Annexin V-FITC/PI Apoptosis Detection Kit was obtained from Yeasen (China). Calcein-AM and propidium iodide (PI) were purchased from Biosharp (China). Anti-Ki67 antibody and anti-HSP70 antibody were purchased from Servicebio (China). Hematoxylin and eosin (H&E) staining kit and TUNEL apoptosis detection kit were obtained from Servicebio (China). All aqueous solutions were prepared using deionized (DI) water purified by a Direct-Q® 3 water purification system (Millipore, USA).

2. **Synthesis of Pdzyme and EGCG-Pdzyme**

Polyvinylpyrrolidone (PVP, Mw = 30 kDa, 133 mg) was first dissolved in 180 mL of methanol under magnetic stirring. Subsequently, 20 mL of an aqueous solution containing 48 mg of palladium chloride (PdCl_2_) was added dropwise to the PVP solution under vigorous stirring. The resulting mixture was allowed to react at 70°C with a stirring speed of 400 rpm for 3 h. After completion of the reaction, excess methanol was removed by rotary evaporation. The remaining solution was subjected to ultrasonication, followed by washing with methanol. The precipitate was collected into centrifuge tubes, and an equal volume of chloroform and fivefold volume of n-hexane were added sequentially. The product was repeatedly washed until neutral pH was achieved. Finally, the obtained Pdzyme was dried in a vacuum oven to yield the final Pdzyme powder.

Pdzyme was dispersed in an aqueous solution of epigallocatechin gallate (EGCG) at a mass ratio of Pdzyme to EGCG of 10:1. The mixture was stirred continuously for 2 h at room temperature to allow surface modification via EGCG adsorption. Subsequently, the dispersion was dialyzed against deionized water to remove unbound EGCG, followed by freeze-drying to obtain EGCG-Pdzyme

3. **Enzymic-mimicking activities measurements**

Catalase (CAT)-Like Activity Assay：The catalase-like activity of Pdzyme and EGCG-Pdzyme was evaluated by monitoring the decomposition of hydrogen peroxide (H₂O₂). Briefly, Pdzyme or EGCG-Pdzyme was dispersed in phosphate-buffered saline (PBS, pH 7.4) at a predetermined concentration. Subsequently, H₂O₂ solution was added to initiate the reaction. The decrease in H₂O₂ concentration was recorded by measuring the dissolved oxygen generation using a dissolved oxygen meter, or alternatively by monitoring the absorbance change of H₂O₂ at 240 nm using a UV–vis spectrophotometer. PBS without nanozyme was used as the control. All measurements were performed at room temperature.

Peroxidase (POD)-Like Activity Assay：The peroxidase-like activity was investigated using 3,3′,5,5′-tetramethylbenzidine (TMB) as a chromogenic substrate. In a typical experiment, Pdzyme or EGCG-Pdzyme was added to an acetate buffer solution (pH 4.0) containing TMB and H₂O₂. The reaction was allowed to proceed for a fixed time period at room temperature. The oxidation of TMB was monitored by measuring the absorbance at 652 nm using a UV–vis spectrophotometer. Control experiments were conducted under identical conditions without nanozyme or H₂O₂ to verify the catalytic role of the nanozyme.

4. **Cell line and cell culture**

The human esophageal squamous cell carcinoma cell line EC109 was obtained from the American Type Culture Collection (ATCC). EC109 cells were cultured in RPMI 1640 medium supplemented with 10% fetal bovine serum (FBS) and 1% penicillin–streptomycin in a humidified incubator at 37 °C with 5% CO₂. Cells were routinely passaged every 2–3 days and harvested using 0.25% trypsin–EDTA at approximately 80–90% confluence. Cells in the logarithmic growth phase were used for all experiments.

5. **Cell viability assessment**

Cell viability was evaluated using the Cell Counting Kit-8 (CCK-8) assay. Briefly, EC109 cells were seeded into 96-well plates at a density of 5 × 10³ cells per well and incubated overnight to allow cell attachment. Subsequently, the cells were treated with different concentrations of Pdzyme or EGCG-Pdzyme for a predetermined period.

For photothermal therapy experiments, cells were exposed to 808 nm near-infrared (NIR) laser irradiation at a specified power density after nanoparticle incubation. After treatment, 10 μL of CCK-8 solution was added to each well and the cells were further incubated at 37 °C for 1–2 h. The absorbance was measured at 450 nm using a microplate reader. Cell viability was calculated as a percentage relative to the untreated control group. All experiments were performed in triplicate.

6. **Hemolysis rate measurement**

The hemolysis assay was performed to evaluate the blood compatibility of Pdzyme and EGCG-Pdzyme. Fresh whole blood was collected from healthy mice and centrifuged at 1500 rpm for 10 min to separate red blood cells (RBCs). The collected RBCs were washed three times with phosphate-buffered saline (PBS, pH 7.4) and diluted to obtain a 2% (v/v) RBC suspension. Pdzyme or EGCG-Pdzyme at different concentrations was incubated with the RBC suspension at 37 °C for 2 h. PBS and deionized water were used as the negative control (0% hemolysis) and positive control (100% hemolysis), respectively. After incubation, the samples were centrifuged at 1500 rpm for 10 min, and the supernatants were collected. The absorbance of the supernatants was measured at 540 nm using a microplate reader.

7. **Reactive Oxygen Species (ROS) Detection**

Intracellular reactive oxygen species (ROS) generation was evaluated using 2’,7’-dichlorodihydrofluorescein diacetate (DCFH-DA) as a fluorescent probe. EC109 cells were seeded in 6-well plates and cultured overnight. After treatment with Pdzyme or EGCG-Pdzyme at indicated concentrations, the cells were exposed to 808 nm near-infrared (NIR) laser irradiation when required.

Following treatment, the cells were washed three times with phosphate-buffered saline (PBS) and incubated with DCFH-DA (10 μM) diluted in serum-free RPMI 1640 medium at 37 °C for 30 min in the dark. After incubation, excess probe was removed by washing with PBS. The fluorescence intensity of intracellular ROS was observed using a fluorescence microscope or quantified by flow cytometry, with excitation at 488 nm and emission at 525 nm.

8. **Apoptosis Assay**

Cell apoptosis was evaluated using an Annexin V-FITC/PI Apoptosis Detection Kit according to the manufacturer’s instructions. EC109 cells were seeded in 6-well plates and cultured overnight. After treatment with Pdzyme or EGCG-Pdzyme at indicated concentrations, the cells were subjected to 808 nm near-infrared (NIR) laser irradiation when required. Following treatment, the cells were collected, washed twice with cold phosphate-buffered saline (PBS), and resuspended in binding buffer. Subsequently, Annexin V-FITC and propidium iodide (PI) were added to the cell suspension and incubated for 15 min at room temperature in the dark. After incubation, the samples were immediately analyzed by flow cytometry.

9. **Animal model**

All animal experiments were approved by the Animal Ethics Committee of the First Hospital of Jilin University (Approval No. JDYY20250927) and conducted under the institutional animal care license **SYXK (Ji) 2024-0022.** The ethical approval was granted on September 8, 2025. All procedures strictly followed the Guide for the Care and Use of Laboratory Animals, with efforts made to minimize animal suffering and ensure animal welfare.EC109 cells cultured in vitro were digested with trypsin, collected, and kept on ice. The cells were resuspended in sterile phosphate-buffered saline (PBS) and mixed with 20% Matrigel to obtain a final cell concentration of 1 × 10⁷ cells/mL. Subsequently, 100 μL of the cell suspension was subcutaneously injected into the right dorsal region of female BALB/c-nu nude mice to establish the tumor-bearing model.

Tumor formation was monitored daily, and the appearance of a palpable bulge at the injection site was regarded as successful tumor establishment. Tumor size was measured using a digital caliper by recording the tumor length and width. Tumor volume (V) was calculated according to the following formula: V = length × width × width / 2

10. **Photothermal ability evaluation in vitro and in vivo**

Photothermal Performance Evaluation In Vitro：The photothermal performance of Pdzyme and EGCG-Pdzyme was evaluated under 808 nm near-infrared (NIR) laser irradiation. Briefly, aqueous dispersions of Pdzyme or EGCG-Pdzyme at different concentrations were placed in quartz cuvettes and irradiated with an 808 nm NIR laser at a specified power density for a fixed duration. During laser irradiation, the temperature changes of the samples were recorded at predetermined time intervals using an infrared thermal imaging camera. Deionized water was used as the control.

To assess the photothermal stability, the samples were subjected to repeated laser on/off cycles, and the temperature variation was recorded to evaluate the reproducibility and stability of the photothermal effect.

Photothermal Performance Evaluation In Vivo：Tumor-bearing BALB/c-nu nude mice were used to evaluate the in vivo photothermal performance. When the tumor volume reached approximately 100 mm³, mice were randomly divided into different treatment groups. Pdzyme or EGCG-Pdzyme was administered via intratumoral injection at a predetermined dose. After injection, the tumor sites were irradiated with an 808 nm NIR laser at a specified power density for a fixed duration.The temperature changes at the tumor sites were continuously monitored using an infrared thermal imaging camera during laser irradiation. Thermal images were captured at designated time points to assess heat generation efficiency. Mice injected with PBS and exposed to laser irradiation were used as controls.

**Photothermal conversion efficiency calculation:** The photothermal conversion efficiency of EGCG-Pdzyme was calculated according to a previously reported method. Briefly, the temperature change of the EGCG-Pdzyme dispersion under continuous NIR laser irradiation was recorded until a steady-state temperature was reached, followed by monitoring the natural cooling process after the laser was turned off. The photothermal conversion efficiency (η) of EGCG-Pdzyme was calculated as follows:

$\eta=\frac{hS\triangle T_{max}-Q_{s}}{I(1-{10}^{-A})}$ (1)

where *h* is the coefficient of heat transfer, S is the container surface area, *ΔT_max_* is the temperature change of the EGCG-Pdzyme solution, *I* is the power of the NIR laser, *A* is the absorbance of the solution of EGCG-Pdzyme at 808 nm, and *Qs* is the heat associated with the light absorbance of the solution.

$hs=\frac{m_{D}C_{D}}{\tau s}$(2)

where m_D_ is the mass of the solution, C_D_ is the specific heat capacity of the system (taken as 4.2 J/(g·°C) for the aqueous solvent), and $\tau_{s}$ is the system time constant.

11. **In vivo toxicity assessment.**

The in vivo biosafety of Pdzyme and EGCG-Pdzyme was systematically evaluated during the treatment period. The body weight of tumor-bearing BALB/c-nu nude mice was recorded every four days to monitor potential systemic toxicity.

At the end of the treatment, mice were euthanized, and major organs, including the heart, liver, spleen, lung, and kidney, were harvested for histological analysis. The collected organs were fixed in 4% paraformaldehyde, embedded in paraffin, sectioned, and stained with hematoxylin and eosin (H&E) for pathological examination.

To further assess hematological and biochemical safety, blood samples were collected for routine blood analysis and serum biochemistry tests, including liver function indicators (ALT and AST) and kidney function indicators (BUN and CREA). The results were compared with those of the control group to evaluate potential organ damage or systemic toxicity.

**12. Statistical analysis**

All experimental data are presented as mean ± standard deviation (SD). Statistical analyses were performed using GraphPad Prism software (GraphPad Software, USA). Comparisons between two groups were conducted using Student’s t-test, while comparisons among multiple groups were performed using one-way analysis of variance (ANOVA) followed by appropriate post hoc tests.

**Figure S1.** Figure S1. Characterization of Pdzyme and EGCG-Pdzyme. (A) XRD patterns of Pdzyme and EGCG-Pdzyme. (B) UV–Vis–NIR absorption spectra of Pdzyme and EGCG-Pdzyme in the range of 200–1000 nm.

**Figure S2.** Time-dependent visual observation of catalytic reactions in ddH₂O, Pdzyme, and EGCG-Pdzyme.

**Figure S3.** pH-dependent and temperature-dependent CAT-like activities of Pdzyme, and EGCG-Pdzyme.

**Figure S4.** POD-like catalytic activity of Pdzyme and EGCG-Pdzyme toward OPD and TMB substrates.

**Figure S5.** Linear fitting of the cooling time versus $-\ln\theta$ for calculating the photothermal conversion efficiency of EGCG-Pdzyme. The fitted time constant ($\tau_{s}$) was 194.91 s, and the photothermal conversion efficiency ($\eta$) was calculated to be 22.1%.

**Figure S6.** Cell viability of LX2, AC16, and HEK293 cells after incubation with EGCG-Pdzyme at different concentrations for 24 and 48 h.

**Figure S7.** Hematological parameters of mice after treatment with Control, Pdzyme, and EGCG-Pdzyme.
